# Molecular innovations in plant TIR-based immunity signaling

**DOI:** 10.1093/plcell/koac035

**Published:** 2022-02-10

**Authors:** Dmitry Lapin, Oliver Johanndrees, Zhongshou Wu, Xin Li, Jane E Parker

**Affiliations:** 1 Department of Plant-Microbe Interactions, Max Planck Institute for Plant Breeding Research, Cologne 50829, Germany; 2 Plant-Microbe Interactions, Department of Biology, Utrecht University, Utrecht 3584 CH, The Netherlands; 3 Michael Smith Labs and Department of Botany, University of British Columbia, Vancouver BC V6T 1Z4, Canada; 4 Cluster of Excellence on Plant Sciences (CEPLAS), Duesseldorf 40225, Germany

## Abstract

A protein domain (Toll and Interleukin-1 receptor [TIR]-like) with homology to animal TIRs mediates immune signaling in prokaryotes and eukaryotes. Here, we present an overview of TIR evolution and the molecular versatility of TIR domains in different protein architectures for host protection against microbial attack. Plant TIR-based signaling emerges as being central to the potentiation and effectiveness of host defenses triggered by intracellular and cell-surface immune receptors. Equally relevant for plant fitness are mechanisms that limit potent TIR signaling in healthy tissues but maintain preparedness for infection. We propose that seed plants evolved a specialized protein module to selectively translate TIR enzymatic activities to defense outputs, overlaying a more general function of TIRs.

## Teaser

Plants have evolved specialized protein modules to connect TIR domain signaling to Ca^2+^ influx and mount effective defense responses.

## Introduction

Eukaryotes and prokaryotes have evolved very different immune systems to transmit the detection of invaders into effective defense responses. Nevertheless, the Toll and interleukin-1 receptor-like (TIR) protein domain of ∼150 amino acids is a shared element of host immunity and cell death programs across kingdoms. The broad taxonomic span and functional significance of TIR sequence homology were recognized at the beginning of the 1990s with the cloning of *Drosophila melanogaster* Toll and tobacco (*Nicotiana tabacum*) N receptors and in comparative studies with the mouse (*Mus musculus*) Interleukin-1L receptor (Sims et al., 1988; Whitham et al., 1994; Lemaitre et al., 1996). TIR domains often exist as fusions with sensor domains that recognize molecules produced by pathogens or the host in response to infection. These molecules include lipopolysaccharides and other pathogen-associated molecular patterns (PAMPs), host-derived interleukin-1 (IL-1) and danger molecules, or variable virulence factors (effector proteins) delivered by pathogens. Most characterized plant TIR-containing proteins are receptors for pathogen effectors (O’Neill and Bowie, 2007; Kawasaki and Kawai, 2014; Morehouse et al., 2020; Tamborski and Krasileva, 2020). Analogous to PAMP-triggered inflammatory responses activated by animal Toll-like receptors, pathogen effector recognition drives plant cells into the transcriptional mobilization of defense pathways, resulting in disease resistance and an alerted state of neighboring cells to subsequent attack (Cui et al., 2015; Betsuyaku et al., 2018).

We will attempt to put together an A to Z (still fragmentary) picture of how plants activate and regulate TIR signaling activity and translate it into transcriptional reprogramming and defense. Because of the remarkable synergy between analyses of TIRs from different groups of organisms, we show the extent and specifics of TIR distribution patterns across the tree of life and bring in examples of nonplant TIR proteins. We further incorporate evolutionary insights that collectively show the extent of molecular innovations in plant TIR signaling and suggest sub-functionalization of TIRs in plants. We conclude that TIR enzymology in the context of TIR evolution and diversity in plants will be crucial to our understanding of TIRs as regulators of plant resilience to biotic and abiotic stress.

## TIR is a versatile protein domain in prokaryotes and eukaryotes

### TIR domain homology is found in many kingdoms of life

**Figure 1:**
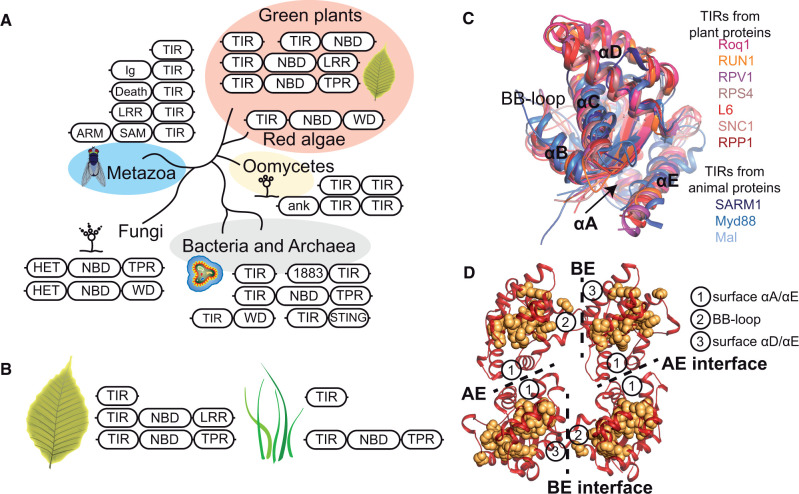
The TIR domain is shared by prokaryotes and eukaryotes. A, Selection of predominant domain architectures in predicted TIR-containing proteins from the indicated taxonomic groups (EBI HMMER, Ensembl genomes with bacteria and archaea grouped together, release 104). Abbreviations of domain names: Death, protein–protein interaction domain originally found in cell death-promoting receptors of tumor necrosis factors (PF00531); SAM, sterile alpha motif, ank, ankyrin repeats; 1883, domain of unknown function DUF1883. Fungi have a TIR-like HET domain in protein architectures with C-terminal WD40 or TPR repeats and a central NBD. B, Dicot and monocot plants differ in TIR protein repertoires; a TNL (TIR-NBARC-LRR) architecture is absent from monocots, many magnoliids, and some groups of dicots. C, Structural alignment of TIRs from RPP1 (PDB:7crc, chain C), Roq1 (7jlx, B), RUN1 (6o0w), RPV1 (5ku7, A), RPS4 (4c6t, B), L6 (3ozi, A), SNC1 (5tec, A), SARM1 (6o0q, A), Myd88 (7ber), and Mal (2ndh). Plant and animal TIRs are in shades of red and blue, respectively. Structural elements (α-helices and the BB-loop between β-strand B and α-helix B) are indicated with letters according to nomenclature. Plant TIRs carry an extended αD helical region. D, Conserved TIR amino acids (yellow spheres) mapped onto the RPP1 TIR tetramer (PDB:7crc, red cartoon). In TNL RPP1, the AE interface is formed by the αA and αE surfaces of individual TIRs, and the BE interface involves the BB-loop of one TIR and the surface between the αD and αE of another. TIRs can also use a DE interface formed by αD and αE surfaces and both TIRs. Conserved positions in TIRs map mainly to the domain core and to surfaces around the catalytic site. Amino acid positions in the hidden Markov model of TIR (PF01582.22) were considered conserved if their information content was ∼2 or higher (https://skylign.org/).

TIR domains (TIRs) are versatile modules that link up with other protein domains in order to transmit signal information. In *Arabidopsis thaliana* (Arabidopsis) alone, TIRs are found in 53 distinct domain architectures (Van de Weyer et al., 2019). However, three principal TIR domain groups are evident across kingdoms: (1) short sequences primarily composed of one or more TIRs, (2) TIRs fused to a repeat or other sensor domain, and (3) TIRs connected to a nucleotide-binding domain (NBD) and C-terminal repeats (Figure 1A).

The first TIR domain protein architecture is present in prokaryotes, oomycetes, plants, and animals. It includes TIR-only proteins and TIR proteins with short additional domains, such as transmembrane and protein–protein interaction regions. Within this group are the well-characterized vertebrate adaptor proteins Myeloid differentiation primary response 88 (Myd88) and MyD88 adaptor-like (Mal; O’Neill and Bowie, 2007), a set of conserved angiosperm TIR-only proteins, and Arabidopsis TIR-only RECOGNITION of HopBA1 (RBA1; Meyers et al., 2002; Nishimura et al., 2017). The TIR-only or TIR+short domain is the most common architecture in plants (Meyers et al., 2002; Johanndrees et al., 2021).

The second protein architecture has TIRs fused to the stimulator of interferon genes (STINGs) domain (Morehouse et al., 2020) or leucine-rich repeat (LRR), immunoglobulin (Ig), and ankyrin repeat regions (Figure 1A). Archetypical representatives of group II TIR proteins are animal LRR-containing Toll-like receptors (TLRs), which detect PAMPs (O’Neill and Bowie, 2007; Kawasaki and Kawai, 2014). Some analyzed oomycetes share uncharacterized ankyrin repeat-TIR proteins that would also fall into this group.

The third TIR protein architecture, found in plants and bacteria, has TIRs attached N-terminally to NBD and LRR, WD40, or tetratricopeptide repeat (TPR) domains (Figure 1A; Sarris et al., 2016; Johanndrees et al., 2021). Plant proteins from this group with the central Apaf1/R/CED4-like NBD (TIR-NBARC-LRR, or TNLs) act as immune receptors that bind pathogen effectors directly or detect their manipulation of host physiology during infection (Monteiro and Nishimura, 2018; Tamborski and Krasileva, 2020). Truncated TIR-NBARCs are common in plants as well (Meyers et al., 2002; Nandety et al., 2013; Johanndrees et al., 2021). Interestingly, an N-terminal HET domain of fungal proteins with central NBD and C-terminal TPR or WD40 repeats involved in incompatibility shares remote similarity to TIRs, including conserved functionally important glutamate (Dyrka et al., 2014; see below; Figure 1A). The broad taxonomic distribution of TIRs and their integration into diverse protein domain architectures underscores the importance of this domain across organisms.

### Similar patterns of TIR evolution in plants and animals

Two contrasting trends in TIR evolution are evident in both plants and animals. One involves high levels of TIR sequence and copy number variation. Arabidopsis TIR regions generally show signatures of diversifying selection (Chae et al., 2014; Van de Weyer et al., 2019), which is consistent with engagement of some Arabidopsis TIRs in the detection of variable pathogen effectors (Nishimura et al., 2017; Guo et al., 2020). Intramolecular interaction of an N-terminal TIR with a C-terminal effector-sensing domain in Arabidopsis TNL receptor RESISTANT TO *RALSTONIA SOLANACEARUM* 1 (RRS1) is crucial for receptor activation by specific effectors in response to bacterial attack (Guo et al., 2020). In the bacterial Thoeris antiphage system, TIRs also likely contribute to the recognition specificity (Ofir et al., 2021). Across plants and animals, genes encoding TIR-containing proteins show high copy number variations among species. TLR numbers can be high in invertebrates but are low in mammals (Buckley and Rast, 2012; Tassia et al., 2017). Similarly, multiple groups of dicots have expanded TNL repertoires (http://compbio.nju.edu.cn/app/ANNA/). However, plants in the order *Caryophyllales* have a reduced TNL set, magnoliids encode zero to few TNLs, and monocots together with multiple aquatic flowering plant species have lost TNLs altogether (Shao et al., 2016; Monteiro and Nishimura, 2018; Lapin et al., 2019; Baggs et al., 2020; Tamborski and Krasileva, 2020; Liu et al., 2021; Wu et al., 2021a; Figure 1B).

Certain TIR groups follow a different trend of sequence evolution in which they exhibit a high degree of conservation and retention across species. For example, TIRs from Myd88/Mal and the regulator of neuronal cell death Sterile Alpha and TIR Motif Containing 1 (SARM1) have maintained sequence identity and low copy number from insects to humans for over ∼800 million years (MY) (http://www.timetree.org/; O’Neill and Bowie, 2007; Kumar et al., 2017; Toshchakov and Neuwald, 2020). Land plants possess conserved TIRs with a broad taxonomic distribution as well. As a case point, TIR-NBARC-TPR (TNP) homologs (also known as XTNX) are present in multiple land plants from bryophytes to angiosperms, indicating they have been conserved for over ∼500 MY (Meyers et al., 2002; Nandety et al., 2013; Zhang et al., 2017b; Johanndrees et al., 2021; http://www.timetree.org/; Kumar et al., 2017, also see the GitHub repository associated with this review: https://github.com/rittersporn/Lapin-etal_PlantCell-review_2022). In some TNP-like proteins, the TPR domain is not detected (Johanndrees et al., 2021).

Another conserved group of TIR-only proteins is present in numerous monocot and dicot plants (Meyers et al., 2002; Nandety et al., 2013; Johanndrees et al., 2021). In this regard, monocots have notably retained TNPs and conserved TIR-only sequences but lost TNLs (Figure 1B; Meyers et al., 2002; Nandety et al., 2013; Shao et al., 2016; Zhang et al., 2017b; Johanndrees et al., 2021; Liu et al., 2021). The in vivo functions of conserved plant TIR-containing proteins are so far unknown. In animals, conserved TIR proteins do not sense pathogen-derived molecules or cytokines directly. Instead, human SARM1 cell death-promoting activity is regulated by small endogenous metabolites that register cellular metabolic status (Figley et al., 2021). Myd88 and Mal act as intracellular adaptors in signal transduction from Toll-like and Interleukin-1 receptors activated outside animal host cells (O’Neill and Bowie, 2007).

Analyses of the amino acid sequence patterns of TIRs revealed >30 subtypes across plants, animals, and prokaryotes, coinciding with functionally defined groups, for example, Myd88 and TLR TIRs (Toshchakov and Neuwald, 2020). This highlights the usefulness of grouping TIRs based on their sequence similarity to predict functional types. Two conserved plant TIR subtypes corresponding to TIRs in TNPs and TIR-only proteins were detected by examining sequence similarity and performing phylogenetic analyses, suggesting a degree of sub-functionalization in plant TIRs as well (Meyers et al., 2002; Nandety et al., 2013; Zhang et al., 2017b; Toshchakov and Neuwald, 2020; Johanndrees et al., 2021).

### Plant TIRs have an extended **ɑ**-helical D region

Comparisons of plant, bacterial, and animal TIR structures revealed that they share a flavodoxin-like ɑ/β-fold in which a central five-stranded β-sheet is surrounded by five **ɑ**-helices (Bayless and Nishimura, 2020; Nimma et al., 2021; Movie 1). These structural elements are named alphabetically to allow comparative studies (Figure 1C). The majority of amino acid positions that are conserved in TIRs form the structural core, which is probably essential for conformational stability (Figure 1D). Despite an overall similarity to animal and bacterial TIRs, structurally characterized plant TIRs have a prominent extended **ɑ**-helical D region (Bernoux et al., 2011; Figure 1C). Mutations in this extended region compromise the cell death-inducing activity of the L6 TIR, Arabidopsis RBA1 and the TNL RESISTANT TO *PSEUDOMONAS SYRINGAE 4* (RPS4), indicating that this molecular innovation has functional relevance (Bernoux et al., 2011; Sohn et al., 2014; Yu et al., 2021). The recently released cryogenic electron microscopy (cryo-EM) structure of TIR from flax (*Linum usitatissimum*) TNL L7 suggests that the **ɑ**-helical D region contributes to the diversification of TIR enzymatic activities in plants (Yu et al., 2021; see section below).

## Uncovering the roles of TIR signaling in plants

### Genes encoding TIR-containing proteins are transcriptionally upregulated in response to PAMPs

**Figure 2:**
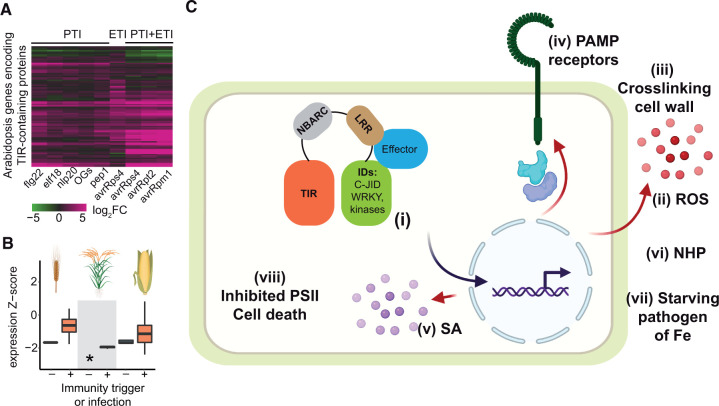
Functions of TIR-containing proteins in plant immunity. A, Genes encoding TIR-containing proteins in Arabidopsis are strongly induced in response to PAMPS and after recognition of thr bacterial effectors avrRpm1 and avrRpt2 by CNLs or avrRps4 by TNLs in accession Col-0. PAMPs administered as pure compounds; avrRps4 is expressed in plant cells without the addition of PAMPS; PTI+ETI, delivery of the indicated effectors mediated by the *Pseudomonas fluorescens* 0-1 EtHAn strain, which also induces PTI. Gene expression data are from (Saile et al., 2020; Bjornson et al., 2021; Ngou et al., 2021). Arabidopsis TIR-containing proteins were predicted using TIR HMM (PF01582.22, *E* < 0.001, HMMER 3.3.1, TAIR10). B, Expression of *TIR-only* genes in monocots is immune trigger-inducible. Expression levels of genes were derived from public RNAseq data for infected or noninfected barley *(Hordeum vulgare, n* = 153 samples, spike on the plot), rice *(Oryza sativa, n* = 117, rice cartoon), or maize (*Zea mays*, *n* = 142, corncob on plot). Expression Z-scores are based on per-sample normalized transcript abundance for all genes to allow comparison between different studies (Johanndrees et al., 2021). * represents no expression detected. C, Cartoon summarizing known TNL ETI outputs in Arabidopsis. TNL receptors often intercept pathogen effectors via IDs (i). Activated TNLs allow for timely transcriptional reprogramming of host cells for defenses associated with apoplastic ROS accumulation (ii) and fortifying the cell wall (iii). Expression of PAMP receptors and proteins involved in early PTI is transcriptionally boosted during TNL ETI (iv). TNL ETI also leads to enhanced accumulation of SA (v) and NHP (vi), which promote local and systemic resistance. TNL pair RRS1-RPS4 mediated ETI is associated with a reduction of soluble iron levels in the apoplast, which helps starve pathogenic Pseudomonas (*Pst*) bacteria (vii). Inhibition of PSII performance is a further signature of RRS1-RPS4 ETI, which promotes chloroplastic ROS accumulation and cell death (Su et al., 2018). The link to source data and code is https://github.com/rittersporn/Lapin-etal_PlantCell-review_2022. Created with BioRender.com.

The transcriptional activation of genes encoding TIR-containing proteins is a conserved feature of immune responses in flowering plants (Nandety et al., 2013; Johanndrees et al., 2021; López-Márquez et al., 2021; Tian et al., 2021). Indeed, in Arabidopsis, these genes are rapidly induced in response to diverse PAMPs and following the recognition of the pathogen effectors by NBARC and LRR-containing receptors (NLRs; Figure 2A; Nandety et al., 2013; Saile et al., 2020; Bjornson et al., 2021; López-Márquez et al., 2021; Ngou et al., 2021; Tian et al., 2021; Yuan et al., 2021b). Even monocots that do not have TNLs display pathogen-triggered expression of conserved *TIR-only* genes (Figure 2B; Nandety et al., 2013; Johanndrees et al., 2021), suggesting that TIRs play a conserved role in bolstering the plant immune response (Tian et al., 2021).

### Plant TIR-domain proteins function as receptors of pathogen effectors

Many characterized plant TIR-containing proteins function as receptors for pathogen effectors to initiate effector-triggered immunity (ETI; Figure 2C). Specific effector recognition is mediated by the TNL LRRs (Krasileva et al., 2010; Steinbrenner et al., 2015; Tamborski and Krasileva, 2020), but additional domains, collectively called integrated domains (IDs), can assist NLR in direct sensing of effectors (Kroj et al., 2016; Sarris et al., 2016). IDs resembling WRKY transcription factors (TFs), zinc finger CCCH TFs, and protein kinases are widespread among TNLs (Figure 2C, section labeled (i)). Functionally, the WRKY ID in Arabidopsis TNL RRS1 enables interception of bacterial effectors as a decoy for their WRKY TF virulence targets (Le Roux et al., 2015; Sarris et al., 2015). The Cryo-EM structures of the effector-activated TNLs RECOGNITION OF *PERONOSPORA PARASITICA 1* (RPP1^WsB^) and Recognition of XopQ 1 (Roq1) reveal an additional C-terminal ID with a jelly-roll and IgG fold (C-JID, PFAM: PF20160), which strengthens LRR-selective effector binding (Krasileva et al., 2010; Steinbrenner et al., 2015; Ma et al., 2020; Martin et al., 2020). C-JID matches the postLRR sequence motifs found in multiple eudicot TNLs (Dodds et al., 2001; Van Ghelder and Esmenjaud, 2016). It is the most common TNL ID domain, being present in ∼50% of TNLs in some plants (Dodds et al., 2001; Van Ghelder and Esmenjaud, 2016; Ma et al., 2020; Saucet et al., 2020; Maruta et al., 2022). Identifying IDs like C-JID provides opportunities to potentially customize TNL recognition specificity.

### Effector-triggered TNLs bolster multiple defense sectors

What then is the purpose of TNL ETI? First, TNL ETI protects and potentiates immunity signaling triggered by PAMPs (PAMP-triggered immunity [PTI]; Figure 2C, ii–iv). One mechanism involves boosting of a PAMP-triggered reactive oxygen species (ROS) burst, as shown in studies with the TNL receptor pair RRS1–RPS4 (Figure 2C, ii). Upon potentiation by ETI, PAMP (flg22) recognition leads to sustained apoplastic ROS accumulation to levels exceeding those induced by PTI or ETI alone (Ngou et al., 2021; Yuan et al., 2021b). Apoplastic ROS (such as H_2_O_2_) can crosslink proteins and polysaccharides in the cell wall, likely to limit pathogen spread (Smirnoff and Arnaud, 2019). A membrane-localized NADPH/respiratory burst oxidase protein D (RBOHD) is the main enzyme for apoplastic ROS production in ETI (Kadota et al., 2019; Ngou et al., 2021; Yuan et al., 2021b). It is also critical for cell wall lignification (Lee et al., 2013), which was found to limit the growth of *Pseudomonas syringae* pv. *tomato* DC3000 (*Pst*) bacteria in ETI mediated by coil-coiled (CC) NLR receptors (CNLs; Lee et al., 2019). Hence, ROS-mediated cell wall fortification might be a general feature of ETI (Lee et al., 2019). Moreover, RRS1-RPS4 ETI transcriptionally induced receptor-like kinases and receptor-like proteins in the absence of PAMPs, providing another clue about how TNLs protect and enhance PTI machineries (Figure 2C, iv; Ngou et al., 2021; Yuan et al., 2021b). To sum up, TNL ETI boosts a PAMP-triggered ROS burst and transcriptionally induces PAMP receptors and immunity components to preserve and amplify anti-pathogen resistance. This action is important for reinstating PTI machineries that are compromised by effector manipulation (Yuan et al., 2021a).

Second, studies using Arabidopsis RRS1-RPS4 showed that TNL ETI boosts signaling by the more ancient hormone salicylic acid (SA), which operates in the defense systems of seed and nonseed land plants (Pieterse et al., 2012; Gimenez-Ibanez et al., 2019; Peng et al., 2021; Figure 2C, v). SA biosynthesis and signaling pathways are vulnerable to manipulation by pathogen effectors (Tanaka et al., 2015). RRS1-RPS4 ETI transcriptionally protects the plant immune system against genetic or pathogenic interference with SA defense (Zhang et al., 2003; Bartsch et al., 2006; Kim et al., 2012; Cui et al., 2015; Mine et al., 2018; Bhandari et al., 2019; Lapin et al., 2020). An important network-level function of TNL ETI is to provide defense routes that do not rely on SA (Cui et al., 2017). One such route involves the synthesis of *N*-hydroxy-pipecolic acid (NHP), which drives local and systemic immunity (Figure 2C, vi; Bartsch et al., 2006; Mishina and Zeier, 2006; Chen et al., 2018; Hartmann et al., 2018).

Third, TNL ETI can help starve pathogens to restrict their growth (Figure 2C, vii). The bacterial effector avrRps4 promotes the accumulation of soluble iron (Fe) in the apoplast of Arabidopsis leaves, which is otherwise nutrient-depleted (Xing et al., 2021). As a countermeasure, avrRps4-activated TNL RRS1-RPS4 signaling reduces apoplastic Fe availability to *Pst* bacteria (Xing et al., 2021). The process of limiting Fe access to *Pst* aligns with the earlier finding that the activation of Arabidopsis NLR immunity reduces the transcriptional upregulation of a bacterial Fe acquisition pathway (Nobori et al., 2018, 2020). RRS1-RPS4 ETI also leads to reduced photosynthetic and photosystem II (PSII) activity and the dampening of photosynthesis-related gene expression (Figure 2C, viii; Su et al., 2018; Saile et al., 2020; Griebel et al., 2021). This physiological dampening helps Arabidopsis leaf cells accumulate ROS in chloroplasts and execute cell death (Su et al., 2018), which is a hallmark of NLR ETI (Cui et al., 2015; Monteiro and Nishimura, 2018). Another possible consequence of reduced PSII activity is the depletion of nutrients available to microbes, which is consistent with a plant strategy to starve invading bacteria, even during PTI (Yamada et al., 2016).

## Many TIRs exhibit tightly regulated NADase activity

### Molecular requirements for TIR NADase activity

**Figure 3:**
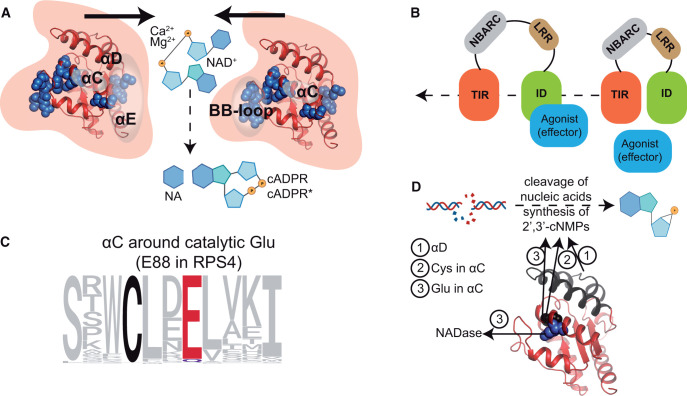
Molecular requirements for NAD^+^ hydrolysis by TIRs. A, Interaction between TIRs via the BB-loop and surface between αD and αE enables the formation of a catalytic site for the hydrolysis of NAD^+^ to NA and cyclic ADP-ribose or a cADPR variant. In the RPP1 resistosome (an effector-induced tetramer), the enzymatic reaction is facilitated by bivalent cations Ca^2+^ and Mg^2+^. RPP1^WsB^ TIR (PDB:7crc, chain C) is shown. Amino acid positions found to be critical for NAD^+^ hydrolytic activity in different plant TIRs (blue spheres) are mapped onto RPP1. B, Pathogen effectors serve as agonists to relieve TIRs from inhibition by other domains (based on studies of TNLs RPP1, Roq1, and RRS1-RPS4) and allow the reactions in (A). C, Profile of the catalytic motif in the αC helix of plant TIRs. The conserved glutamate (E) residue essential for NAD* hydrolysis corresponds to Glu88 in TNL RPS4 (red). A neighboring conserved cysteine (black) is important for the cleavage of nucleic acids and synthesis of 2′,3′-cNMPs. D, While a conserved glutamate in αC (blue) is critical for both NADase and cNMP synthetase activities, mutations in αD and the conserved cysteine in αC (black) specifically impair the latter. RPP1^WsB^ TIR (PDB:7crc, chain C) was used as a template for consistency. Created with BioRender.com.

A breakthrough in understanding TIR functions came with the discovery that human (*Homo sapiens*) SARM1 exhibits nicotinamide (NA) adenine dinucleotide (NAD^+^) hydrolyzing activity (Essuman et al., 2017), followed by similar findings for prokaryotic and plant TIRs (Essuman et al., 2018; Horsefield et al., 2019; Wan et al., 2019; Morehouse et al., 2020; Eastman et al., 2021). This enzymatic activity appears to be a common TIR feature, although it was not found in the mammalian TLR2 or TIR adaptor proteins examined (Horsefield et al., 2019; Bayless and Nishimura, 2020). SARM1 TIR cleaves NAD^+^ into NA and cyclic ADP-ribose (cADPR) or ADPR (Essuman et al., 2017). NAD^+^ hydrolysis to cADPR or ADPR variants by plant TIRs was detected both in vitro with purified proteins and in vivo, demonstrating that these TIR domains can operate as autonomous enzymes (Horsefield et al., 2019; Wan et al., 2019; Duxbury et al., 2020; Ma et al., 2020; Ofir et al., 2021; Figure 3). However, the identity of physiological substrate(s) and product(s) of TIR NADase enzymes remains an unresolved issue in plant immunology.

Several reaction parameters influence TIR-mediated NAD^+^ hydrolysis (Figure 3A). First, TIR NADase activity requires the proximity of at least two TIR domains (Figure 3A, arrows pointing to each other). Experimentally, proximity can be achieved by adding solution-crowding agents such as polyethyleneglycol (Horsefield et al., 2019) or by inducing oligomerization of a chimera between an animal NLR and a plant TIR through binding of a PAMP (Duxbury et al., 2020). In the case of a full-length TNL, TIRs are brought together as two asymmetrically aligned TIR pairs via effector-induced TNL tetramerization, as shown for TNLs RPP1^WsB^ and Roq1 (Ma et al., 2020; Martin et al., 2020; Movie 1). Similarly, recognition of a cyclic dinucleotide by the bacterial STING stimulates NAD^+^ hydrolysis by adjacent TIRs (Morehouse et al., 2020).

Second, upon activation, TIRs are relieved from molecular inhibition by other domains in a full-length protein (Figure 3B). For example, TIRs of human SARM1 are kept apart in a homo-octameric complex in which the orientation of armadillo motif (ARM) oligomerization domains prevents TIRs from interacting with each other (Sporny et al., 2020; Figley et al., 2021; Shen et al., 2021). Peptide interference with the ARM–TIR interaction renders SARM1 autoactive (Shen et al., 2021). NA mononucleotide outcompetes NAD^+^ in the ARMs of SARM1 and thereby likely triggers conformational changes, allowing TIR–TIR interactions (Sporny et al., 2020; Figley et al., 2021). In the Arabidopsis TNL receptor complex RRS1–RPS4, bacterial effectors likely disrupt a self-inhibited RRS1–RPS4 state (Guo et al., 2020) and allow RPS4 TIRs to interact, leading to NAD^+^ hydrolytic activity (Williams et al., 2014; Wan et al., 2019; Duxbury et al., 2020).

Third, TIRs engage in functional interactions via conserved TIR structural elements (Figure 3A). In the TNLs RPP1^WsB^ and Roq1, the most prominent features are a BB-loop of one protomer that fits under the surface formed by the αD and αE of another TIR (“BE” interface) and the “AE” interface formed by αA and αE (Horsefield et al., 2019; Wan et al., 2019; Ma et al., 2020; Burdett et al., 2021; Nimma et al., 2021; Figure 3A; Movie 1). The BE interface-mediated interaction creates an active NADase site in TNL RPP1 and other TIRs (Ma et al., 2020; Martin et al., 2020; Burdett et al., 2021; Nimma et al., 2021).

Fourth, TIR NAD^+^ hydrolytic activity requires a conserved surface-exposed glutamate residue in α-helix C that forms part of the catalytic site (Figure 3, A and C). Mutating this glutamate abolished TIR NAD^+^ hydrolysis and cell death-promoting activity in plant transient expression assays (Krasileva et al., 2010; Sohn et al., 2014; Essuman et al., 2017; Horsefield et al., 2019; Wan et al., 2019; Ma et al., 2020; Martin et al., 2020; Eastman et al., 2021).

Finally, as demonstrated in in vitro assays, the rate of NAD^+^ hydrolysis by plant TNL RPP1^WsB^ is stimulated by the bivalent cations Ca^2+^ and Mg^2+^ (Ma et al., 2020; Figure 3A).

### Emerging NADase-independent properties of TIR cooperative assembly

Not all TIRs contain the conserved catalytic glutamate or exhibit detectable NAD^+^ hydrolysis (Horsefield et al., 2019; Bayless and Nishimura, 2020). Nevertheless, TIR–TIR association is still required for their functions. Mammalian Myd88 and Mal adaptors can form large filaments in vitro via homotypic and heterotypic interactions requiring the BB-loop and other interfaces (Ve et al., 2017; Clabbers et al., 2021; Nimma et al., 2021). Interaction of TLR4 with Mal creates a surface for association with Myd88 (Clabbers et al., 2021), which can then activate IL-1 receptor-associated kinase and the TF Nuclear Factor-κB (NF-κB), thereby driving immune-related transcriptional reprogramming. Bacterial and viral TIR effector proteins can interfere with the TLR:Mal:Myd88 assemblies and thus disarm the immune system (Nanson et al., 2020). The emerging properties of TIR–TIR assemblies led to the model of signaling via cooperative assembly formation (SCAF; Nanson et al., 2020; Nimma et al., 2021). One likely outcome of SCAF is to concentrate signaling molecules and biochemical processes in a subcellular compartment (Ve et al., 2017; Nanson et al., 2020; Clabbers et al., 2021).

Importantly, evidence for the SCAF model for plant TIRs (Nishimura et al., 2017; Zhang et al., 2017a) was found experimentally through cryo-EM analysis of L7 TIR in complex with DNA (Yu et al., 2021; Figure 3D). A structure-guided study revealed that plant TIRs can cleave nucleic acids and synthesize 2′,3′-cyclic nucleotide monophosphates (cNMPs) to promote cell death. TIR 2′,3′-cNMP synthetase and NADase activities have different requirements (Yu et al., 2021; Figure 3D). First, 2′,3′-cNMP synthesis from DNA was detected in L7 TIR filaments using liquid chromatography coupled with mass spectrometry, but NAD^+^ hydrolysis detected by liquid chromatography was most prominent in lower molecular weight L7 TIR fractions (Yu et al., 2021). Second, mutations in a cysteine neighboring the catalytic glutamate and the extended αD helix interfered primarily with 2′,3′-cNMP synthetase activity (Figure 3, C and D). Third, TIR oligomers that assemble via AE and BE interfaces in the RPP1 and Roq1 resistosomes act as NADases. In contrast, TIR oligomerization mediated by AE and DE interfaces is critical for nucleic acid cleavage and 2′,3′-cNMP synthesis (Ma et al., 2020; Martin et al., 2020; Yu et al., 2021). Taken together, nucleic acid cleavage and 2′,3′-cNMP synthetase activity are emerging properties of plant TIR SCAF.

## Plant-specific protein modules translate TIR activity to defense

### Plant TIR immunity signaling branches

**Figure 4:**
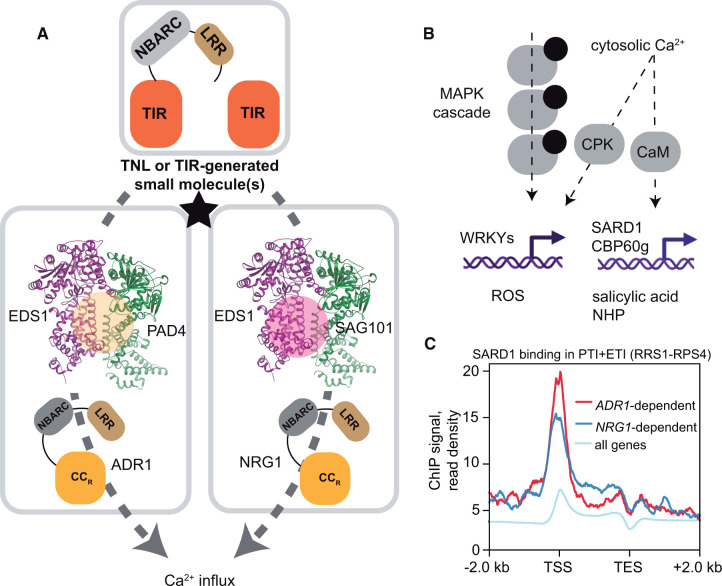
Helper NLR-facilitated Ca^2+^ influx provides a way to transcriptionally reprogram plant cells for defense in TNL ETI. A, Schematic diagram of signal transduction from activated TNLs or TIRs leading to Ca^2+^ influx via the specific cooperation between EDS1 family proteins and helper NLR subgroups. In TNL-activated tissues, dimers of EDS1 with its sequence-related proteins PAD4 or SAG101 engage in association with ADR1 or NRG1 RNLs, respectively. Black star indicates a small transduction of TNL activation into transcriptional reprogramming. Reorganized CC_R_ domains in an activated RNL oligomer are proposed to form a Ca^2+^-permeable membrane-associated ion channel. B, Model depicting the transduction of TNL activation into transcriptional reprogramming. In Arabidopsis, increased cytosolic Ca^2+^ levels activate CPKs, which phosphorylate the TF WRKY48, thereby promoting the expression of RBOHD, which produces apoplastic ROS. CaM are Ca^2+^ receptors inside cells that enable activation of the CaM-binding TF CBP60g. CBP60g and the CBP60g-like TF SARD1 (lacking CaM-binding capacity) transcriptionally promote the biosynthesis of SA and NHP in Arabidopsis*.* Phosphorylation of MPKs 3/6 during TNL ETI leads to the phosphorylation of WRKY TFs, which mobilize immunity gene expression and promote ROS accumulation in tobacco. C, The binding of Arabidopsis SARD1 is enriched at the promoters of genes that are upregulated during RRS1-RPS4 ETI in an ADR1-and NRG1-dependent manner (log2FC ≥ 1, *P*_adj_ ≤ 0.05). Chromatin immunoprecipitation (ChIP) data are from Sun et al. (2015), and information on helper NLR dependency is from (Saile et al., 2020). TSS, transcription start site, TES, transcription end site; all genes, all TAIR10 genes. Processed ChIP data are from (Griebel et al., 2021). The link to source data and code are provided at https://github.com/rittersporn/Lapin-etal_PlantCell-review_2022. Created with BioRender.com.

Immunity outputs from TNLs and TIRs in plants depend on a small group of conserved NLRs that possess a phylogenetically distinct N-terminal CC domain. This domain was originally found in Arabidopsis membrane-associated protein RESISTANCE TO POWDERY MILDEW 8 and is therefore called CC_R_ (Figure 4A; Xiao et al., 2001; Jubic et al., 2019; Feehan et al., 2020). Because CC_R_ NLRs (RNLs) function downstream of pathogen detection, they are also referred to as helper or signaling NLRs. RNLs are specific to seed plants and have an NBARC domain that is phylogenetically different from that of other NLRs (Shao et al., 2016). A role for these NLRs in signal transduction is reflected in their conservation across seed plants and low sequence variation within Arabidopsis (Shao et al., 2016; Monteiro and Nishimura, 2018; Jubic et al., 2019; Van de Weyer et al., 2019). The RNL family can be further divided into the related N REQUIREMENT GENE 1 (NRG1) and ACTIVATED DISEASE RESISTANCE 1 (ADR1) subgroups. While the NRG1 and ADR1 subgroups can partially compensate for each other in TNL-mediated transcriptional reprogramming and pathogen resistance (Castel et al., 2019; Wu et al., 2019; Saile et al., 2020; Sun et al., 2021), genetic and molecular evidence indicates they are operationally distinct (Figure 4A). For example, in RRS1–RPS4 ETI, NRG1s are required for host cell death, whereas ADR1s function predominantly in transcriptional reprogramming of defense genes and pathogen resistance (Bonardi et al., 2011; Castel et al., 2019; Lapin et al., 2019; Wu et al., 2019; Saile et al., 2020; Sun et al., 2021). In Arabidopsis, NRG1s and ADR1s are engaged to different extents downstream of TIRs or TNLs, while in tobacco species *N. tabacum* and *Nicotiana* *benthamiana*, TIRs and TNLs signal primarily through NRG1 (Peart et al., 2005; Qi et al., 2018; Castel et al., 2019; Lapin et al., 2019, 2020; Wu et al., 2019).

Plants have also evolved a small, conserved family of lipase-like proteins for immunity signaling downstream of TNLs and TIRs. These proteins, ENHANCED DISEASE SUSCEPTIBILITY 1 (EDS1), PHYTOALEXIN DEFICIENT 4 (PAD4), and SENESCENCE-ASSOCIATED GENE 101 (SAG101), are collectively referred to as the EDS1 family (Figure 4A; Lapin et al., 2020). The EDS1 family possesses a unique structure in which an N-terminal α/β hydrolase (lipase-like) domain is fused with a C-terminal α-helical bundle “EP” domain (Wagner et al., 2013). PAD4 and SAG101 form mutually exclusive heterodimers with EDS1, which appear to be the minimal functional units for EDS1-dependent defense against pathogens (Wagner et al., 2013; Voss et al., 2019; Dongus and Parker, 2021).

Analyses of Arabidopsis combinatorial mutants have helped to clarify functional relationships between RNLs and EDS1 family members in TNL immunity (Sun et al., 2021; Wu et al., 2021b). Mutants with combinations of defective genes for EDS1 and RNL family members showed differences in signaling outputs of the TNLs SUPPRESSOR OF *NONEXPRESSOR OF PATHOGENESIS-RELATED GENES 1-1*, CONSTITUTIVE 1 (SNC1) and RRS1-RPS4. These differences are consistent with the co-functions of EDS1-SAG101 with NRG1s and of EDS1-PAD4 with ADR1s in defense promotion (Figure 4A). Importantly, elements of the two modules are not functionally interchangeable (Sun et al., 2021; Wu et al., 2021b), even in an SA-deficient background (Sun et al., 2021). Tight genetic cooperation between EDS1-SAG101 and NRG1 was also observed in TNL Roq1-triggered pathogen resistance and host cell death in the wild tobacco *N.* *benthamiana* (Qi et al., 2018; Gantner et al., 2019; Lapin et al., 2019). Notably, cell death responses triggered by various TNLs or TIRs transiently expressed in the tobacco system recruited native or ectopically expressed Arabidopsis EDS1 and SAG101 with NRG1, but not PAD4 or an ADR1 family member (Gantner et al., 2019; Lapin et al., 2019). The co-occurrence of *SAG101* with *NRG1* and *PAD4* with *ADR1* genes in seed plant genomes further supports co-functions between specific helper NLR subgroups and EDS1 dimers in TNL immunity (Collier et al., 2011; Lapin et al., 2019, 2020; Baggs et al., 2020; Liu et al., 2021). Molecularly, the functional cooperation appears to manifest as specific complex formation between Arabidopsis EDS1-SAG101 with NRG1s and EDS1-PAD4 with ADR1s in Arabidopsis and wild tobacco TNL-activated leaf tissues (Sun et al., 2021; Wu et al., 2021c) or upon transient expression of the Arabidopsis TIR-only protein RBA1 in wild tobacco (Wu et al., 2021c).

What underlies the selectivity in complex formation between EDS1 family heterodimers and particular RNL subgroups has not been resolved, although distinctive sequence features in the respective dimer EP domain cavities are probably important determinants (Gantner et al., 2019; Lapin et al., 2019; Sun et al., 2021). We speculate that this pathway choice in TIR signaling provides resilience against interference by effectors. Thus, TIR downstream signaling involves induced complex formation between EDS1 dimers and RNLs to mobilize host defense and cell death machineries when pathogen attack is registered. It remains unclear why Arabidopsis utilizes both modules made of EDS1 dimers and RNLs, whereas wild tobacco only uses EDS1-SAG101 with NRG1. One possible explanation is that the EDS1–PAD4–ADR1s node has broader usage in mobilizing pathways initiated by NLRs and certain cell surface receptors, as observed in Arabidopsis (Dongus and Parker, 2021; Pruitt et al., 2021; Tian et al., 2021). It will be interesting to test whether this idea is borne out in other seed plant lineages, especially monocots, which retained *EDS1*, *PAD4*, and *ADR1* genes but have lost TNLs, *SAG101*, and *NRG1* (Baggs et al., 2020; Lapin et al., 2020).

Studies suggest a degree of selectivity in translating TIR enzymatic activity to defense promotion at the level of the EDS1 family. The TIR of the bacterial disease-promoting effector HopAM1 produces a variant of cADPR, but its cell death activity in wild tobacco is independent of EDS1 (Eastman et al., 2021). Similarly, cell death triggered by human SARM1 TIR and even a maize TNP is *EDS1-*independent (Horsefield et al., 2019; Johanndrees et al., 2021). It is significant that the Arabidopsis phosphodiesterase enzyme NUDIX HYDROLASE HOMOLOG 7 (NUDT7) can cleave 2′,3′-cAMP and 2′,3′-cGMP, suppressing RBA1-mediated cell death in wild tobacco (Yu et al., 2021). Moreover, an Arabidopsis *nudt7* mutation leads to spontaneous *EDS1*-dependent cell death (Bartsch et al., 2006; Straus et al., 2010). Hence, it is likely that an EDS1 dimer–RNL module becomes activated by 2′,3′-cAMP and 2′,3′-cGMP or products derived by their processing during defense amplification, although contributions from other TIR-generated molecules cannot be ruled out (Figure 4A).

### Autoactive NRG1 forms a putative membrane cation channel

Cryo-EM structure-guided studies of CNL HopZ-ACTIVATED RESISTANCE 1 (ZAR1) established that the effector-activated receptor forms a pentameric wheel in which five ZAR1 CC domains become exposed to create a membrane-localized Ca^2+^-permeable channel (Wang et al., 2019; Bi et al., 2021). ZAR1 channel activity requires conserved negatively charged amino acids on the inner side of a CC α-helical funnel (Wang et al., 2019; Bi et al., 2021). ZAR1 structure-guided and sequence-based alignments showed that the N-terminal CC_R_ domains of ADR1 and NRG1 have α-helical amino acid coordinates for a similar oligomer pore or channel (Jubic et al., 2019; Bi et al., 2021; Jacob et al., 2021; Sun et al., 2021). Indeed, mutations in glutamic acid residues Glu14 and Glu27 at the predicted α-helical inner pore of Arabidopsis NRG1.1 disabled Roq1-mediated cell death and resistance in wild tobacco (Sun et al., 2021). A ∼1 MDa Arabidopsis autoactive NRG1.1 complex localized to the plasma membrane when expressed in wild tobacco and caused Ca^2+^ influx in human HeLa cells in the absence of other plant proteins, suggesting that NRG1 exhibits autonomous ion channel activity or that NRG1 perturbs the membrane, leading to Ca^2+^ influx (Jacob et al., 2021). Ca^2+^ influx was also detected for ADR1 and was suppressed by mutating the negatively charged amino acids in its N-terminal CC_R_ domain (Jacob et al., 2021). Hence, CNL and TNL signaling might share the property of increasing Ca^2+^ levels in the cytoplasm (Figure 4A). Such a scheme is supported by the finding that the cell death-inducing activities of CNLs, TNLs, and RNLs were suppressed by treatment with the Ca^2+^ channel blockers LaCl_3_ and GdCl_3_ (Grant et al., 2000; Ma et al., 2008; Jacob et al., 2021). Since a TNL-induced interaction between NRG1 and EDS1-SAG101 was not affected by the CC_R_ funnel mutations, NRG1 Ca^2+^ channel activity promoting TNL immunity and host cell death was placed downstream of the association of NRG1 with EDS1-SAG101 (Jacob et al., 2021; Sun et al., 2021).

Taken together, the above studies suggest a model in which an effector-activated TNL resistosome or self-associating TIRs produce one or more small molecules that promote associations between specific RNLs and EDS1 family heterodimers. The formation of an EDS1 dimer–RNL complex must permit the RNL to function either as an oligomeric pore or Ca^2+^ influx channel or in some other capacity to amplify Ca^2+^ dependent cascades driving transcriptional defense (Figure 4A). As in vitro NADase RPP1^WsB^ activity is enhanced by Ca^2+^ and Mg^2+^ (Figure 3A; Ma et al., 2020), Ca^2+^ influx might in principle further amplify TNL activity in a feed-forward loop.

### Transcriptional activation of plant defense during ETI

A number of Ca^2+^-dependent protein kinases (CPKs) and Ca^2+^/calmodulin (CaM)-regulated TFs contribute to nuclear transcriptional changes during ETI (Tsuda and Somssich, 2015). Several Arabidopsis CPKs, most prominently CPK5 and CPK6, contribute to ETI mediated by the TNL pair RRS1–RPS4 and two CNLs. The phosphorylation of WRKY48 by CPK5 enhances both the DNA binding strength of this TF and the expression of ROS-producing RBOHD (Figure 4B; Gao et al., 2013).

Operating in parallel to CPKs, mitogen-activated protein kinase (MAPK) cascades also transduce signals during ETI (Figure 4B; Tsuda and Somssich, 2015). MAPKs MPK3 and 6 are required for full resistance mediated by the TNLs RRS1-RPS4 in Arabidopsis and N in tobacco (Jin et al., 2003; Adachi et al., 2015; Su et al., 2018). The activation of NLRs is accompanied by the sustained phosphorylation of MPK3 and 6 (Tsuda et al., 2013; Cui et al., 2015, 2017; Su et al., 2018), a process likely related to increased phosphorylation of coreceptors for PAMP sensing receptors (Ngou et al., 2021; Yuan et al., 2021b). The phosphorylation of WRKYs by MPK3/6 induces *RBOHD* expression, an ensuing ROS burst, and host cell death in wild tobacco (Ishihama et al., 2011; Adachi et al., 2015). From the existing data, it seems likely that activated TNLs signal through both CPK and MAPK cascades to increase the expression of immunity executors (e.g. *RBOHD*) via phosphorylation of WRKY TFs (Tsuda and Somssich, 2015; Figure 4B).

Researchers have identified another branch of transcriptional control of TNL ETI involving CaM and CaM-related proteins (Figure 4B). CaMs are conserved Ca^2+^ receptors that interact with proteins after a Ca^2+^/CaM-controlled allosteric change (Kang et al., 2006). The TF CaM-BINDING PROTEIN 60-Like G (CBP60g; Wang et al., 2009) and its homolog SYSTEMIC ACQUIRED RESISTANCE DEFICIENT 1 (SARD1), which lacks a CaM-binding domain (Wang et al., 2011), are prominent regulators of local and systemic resistance in Arabidopsis (Zhang et al., 2010; Wang et al., 2011). CBP60g and SARD1 bind to promoters and induce the expression of defense-related genes encoding the EDS1 family, RNLs, and components of SA and NHP biosynthesis (Zhang et al., 2010; Wang et al., 2011; Sun et al., 2015; Ding et al., 2020). Similarly, SARD1 and CBP60g binding is enriched at genes induced during TNL RRS1-RPS4 transcriptional reprogramming (Figure 4C; Sun et al., 2015; Saile et al., 2020; Griebel et al., 2021). Thus, Ca^2+^ influx during ETI (RRS1-RPS4) is likely transduced by CPKs and CaM into transcriptional defense responses (Figure 4B). The large number of CaM and CaM-like proteins in plant genomes leaves open the question of how Ca^2+^ influx is interpreted by cells during TNL ETI.

## How plants keep TNLs in check

The mis-activation or ectopic expression of TIR-containing proteins is linked to stunting, macroscopic cell death, and sensitivity to low temperatures and osmotic stress (Palma et al., 2007; Gloggnitzer et al., 2014; van Wersch et al., 2016; Ariga et al., 2017). It is thought that TNL and TIR expression levels need to be sufficient for a timely immune response against pathogens but low enough to avoid growth penalties in unchallenged plants. Below, we review mechanisms implicated in the control of TNL expression at the chromatin and posttranscriptional levels in plants (mainly Arabidopsis).

### Four pathways keep basal TNL transcript levels low

**Figure 5:**
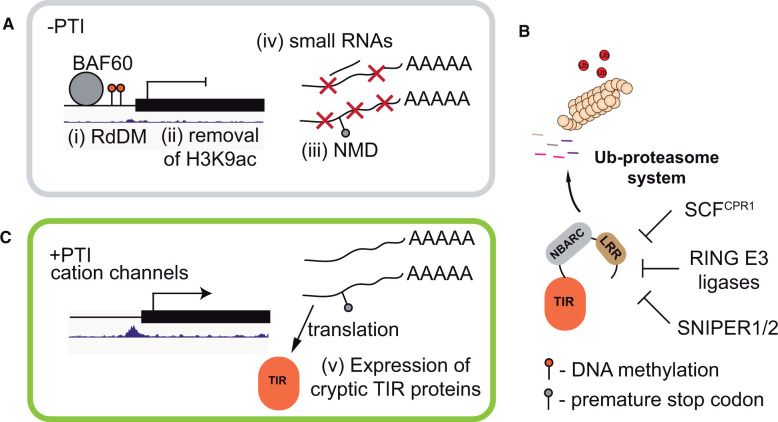
Mechanisms to avoid aberrant TIR/TNL activity in Arabidopsis*.* A, In the absence of pathogen attack, TIR/TNL-encoding genes are suppressed by (i) the RdDM and (ii) via removal of the transcription-permissive acetylation mark on histone H3 at Lys9 (H3K9ac). (iii) NMD depletes aberrant TIR/TNL transcripts produced as a result of poly(A) site selection. (iv) Small RNAs (microRNA and phasiRNA) also help reduce TNL transcript abundance (iv). B, At the posttranslational level, steady-state TNL abundance is regulated by the UPS. Three E3 ligases target TNLs for degradation, with conserved SNIPER1/2 having a broad NLR target range. C, Upon PAMP recognition, the inhibition of NMD and small RNA pathways promotes TIR/TNL expression. Increased expression of TIR/TNL genes is partially dependent on the cation channels. Reduced efficiency of NMD might also help unmask TNL cryptic variation (v) via translation of TIR-only and truncated TNL forms. In (A) and (B), chromatin accessibility profiles are shown for the TNL gene AT4G11170 (Ding et al., 2021; Tian et al., 2021).

One pathway to control basal TNL gene expression in Arabidopsis involves DNA methylation via the RNA-directed DNA methylation (RdDM) pathway (Figure 5A, I; Table 1). In the TNL gene *RESISTANCE METHYLATED GENE 1* (*RMG1*), this is facilitated by a transposable element-like sequence residing in the promoter (Yu et al., 2013; Halter et al., 2021). It is likely that RdDM-mediated suppression of TNL promoter activity is further supported by the chromatin remodeling protein BAF60/SWP73A: this protein suppresses TNL gene expression and can bind the histone mark H3K9me2 (histone H3 dimethylated at Lys9; Huang et al., 2021), which is known to enforce RdDM (Du et al., 2014; Johnson et al., 2014; Li et al., 2018; Figure 5A, i).

**Table 1:** Pathways limiting basal expression of genes encoding TIR-containing proteins in Arabidopsis

Name of mechanism	Brief description	References
RNA-directed DNA methylation	Plant-specific DNA methylation system where CHG and CHH DNA methyltransferases are recruited to chromatin via small and long noncoding RNAs.	Dowen et al. (2012); Yu et al. (2013); Halter et al. (2021); Huang et al. (2021)
Regulation of histone H3 acetylation at Lys9 (H3K9ac)	The histone modification H3K9ac correlates with active transcription at the locus.	Yang et al. (2020)
NMD	Eukaryotic system that degrades transcripts with a premature stop codon, although other types of targets are known.	Gloggnitzer et al. (2014); Jung et al. (2020); Raxwal et al. (2020)
Interference via small RNAs	The mechanism of interference involves the binding of small RNAs to transcripts, initiating their degradation. In the nucleus, small RNAs are also involved in regulating RdDM.	Shivaprasad et al. (2012); Boccara et al. (2014); Zhang et al. (2016); Cai et al. (2018); López-Márquez et al. (2021)
UPS	Eukaryotic protein degradation system linking ubiquitination of a protein substrate to the 26S proteasome. The E3 ubiquitin ligases largely determine substrate specificity.	Cheng et al. (2011); Gou et al. (2012); Dong et al. (2018); Copeland and Li (2019); Zhang et al. (2019); Wu et al. (2020)

Another route to prevent TNL misexpression involves the removal of H3K9ac (transcription-permissive acetylation of H3 at Lys9) at TNL loci in healthy tissues (Figure 5A, ii; Table 1). In Arabidopsis, HISTONE DEACETYLASE 9 (HDA9) and the WD40 repeat protein HIGH EXPRESSION OF OSMOTICALLY RESPONSIVE GENES 15 (HOS15) bind to the promoters of the RNL gene *ADR1-L2* and selected TNL genes and help to deplete H3K9ac at NLR loci (Yang et al., 2020). Accordingly, Arabidopsis *hda19* and *hos15* mutants exhibited transcriptional upregulation of approximately one-third of the NLR gene repertoire, including TNLs and RNLs (Yang et al., 2020).

The third mechanism to control TNL transcript abundance is nonsense-mediated mRNA decay (NMD), which removes aberrant TNL transcripts with premature stop codons (Table 1). NMD keeps basal TNL gene expression levels low and prevents *TNL* and *EDS1*-dependent growth penalties in Arabidopsis (Gloggnitzer et al., 2014; Jung et al., 2020; Raxwal et al., 2020). One source of premature stop codons is the selection of alternative polyadenylation sites in genes. Mass spectrometry and long-read mRNA sequencing identified the RNA-binding protein FPA as a regulator of proximal poly(A) site selection in Arabidopsis, with NLR transcripts being the primary targets (Parker et al., 2021). While these events mostly result in TNL transcripts lacking stop codons, truncated TNL transcripts with premature stop codons and putative *TIR-only* transcripts predicted to be NMD-targeted have also been detected (Parker et al., 2021).

A fourth mechanism to reduce the abundance of TNL transcripts involves small RNAs (Table 1). Small RNAs in a range of plant species show a remarkable match of 22 nucleotide (nt) microRNAs and 21-nt phased secondary small interfering RNAs (phasiRNAs) to NLR gene family members, including TNLs (Zhang et al., 2016; López-Márquez et al., 2021). Most small RNAs correspond to conserved and functionally important amino acid motifs: the P-loop in NBARC and the α-helix in TIR domains (Zhang et al., 2016; López-Márquez et al., 2021; Figure 1C). In Arabidopsis, the TNL gene *MICRORNA-SILENCED TNL1* is targeted by miR825-5p to produce phasiRNAs, triggering the cleavage of numerous other TNL transcripts (López-Márquez et al., 2021). Therefore, small RNAs provide an effective posttranscriptional mechanism for limiting basal TIR/TNL gene expression (Figure 5A, iv).

### Permissive TNL promoter activity contributes to TNL-mediated resistance

Evidence suggests that maintaining basal transcription-permissive chromatin environment is crucial for TNL-mediated resistance. A forward genetic screen for suppressors of *snc1*-associated dwarfism in Arabidopsis identified a plant-specific protein with no known domains: MODIFIER OF SNC1, 9 (MOS9). MOS9 associates with the H3K4 methyltransferase TRITHORAX-RELATED 7 and helps maintain a transcription-correlated H3 Lys4 trimethylation mark at the promoters of TNL-encoding *RPP4* and *SNC1* and a basal level of their transcription (Xia et al., 2013; Leng et al., 2020). Since the *mos9* mutant is defective in RPP4 resistance (Xia et al., 2013), balancing transcription-permissiveness at *TNL* chromatin likely enables plants to respond in a timely manner to pathogen infection.

### E3 ligases limit TNL protein accumulation

Studies of autoimmunity of the TNL mutant *snc1* revealed that the conserved eukaryotic ubiquitin-proteasome system (UPS) regulates TNL protein homeostasis (Copeland and Li, 2019; Table 1). UPS specificity is controlled by E3 ligases that attach ubiquitin to protein targets. Three E3 ligase groups are known to regulate TNL protein levels (Figure 5B). The first two are exemplified by the SKP1-cullin1-F-box (SCF) E3 ligase complex with the subunit CONSTITUTIVE EXPRESSER OF *PATHOGENESIS-RELATED* GENES 1 (CPR1), RING-type E3 ligases Mutant *snc1*-enhancing 1 and 2, and Ubiquitin Protein Ligase E3 Component N-Recognin 7. These E3 ubiquitin ligases appear to have a narrow range of TNL targets (Cheng et al., 2011; Gou et al., 2012; Dong et al., 2018; Zhang et al., 2019). Interestingly, the E3 ligase SCF^CPR1^ requires proteins with the conserved domain Tumor necrosis factor Receptor (TNFR)-Associated Factor (TRAF) to remove excess TNL SNC1 protein (Huang et al., 2016). In animals, TRAF proteins serve as scaffolds or E3 ligases in TLR and TNFR signaling (Yang and Sun, 2015; Park, 2018). A component of the general chaperone machinery, heat shock protein HSP90.3, also participates in the assembly and functioning of E3 ubiquitin ligase complexes that control the steady-state levels of some TNLs (Copeland and Li, 2019; Liang et al., 2020). The third E3 ligase group includes Arabidopsis RING class proteins *snc1*-influencing plant E3 ligase reverse 1 (SNIPER1) and SNIPER2, which control the turnover of multiple TNL and CNL proteins (Wu et al., 2020; Figure 5B). In contrast to most E3 ligases, SNIPERs are conserved in dicot plants, suggesting they play a role in regulating NLR accumulation across species (Wu et al., 2020). Since the *SNIPER1* gene is bound by SARD1 and is induced during PTI and ETI, SNIPER1 and probably other E3 ligases are thought to help deactivate immune responses and reduce the physiological costs of defense (Wu et al., 2020).

## PAMP perception removes the brakes on TNL gene expression

How TIR/TNL gene expression is turned up during infection is poorly understood mechanistically, but PAMP perception plays an important role in this process. This induction is probably facilitated by active cation channels (Bjornson et al., 2021; Ngou et al., 2021; Tian et al., 2021; Yuan et al., 2021b) and is associated with a more open chromatin state at promoter regions (Ding et al., 2021) (Figures 2A and 5C). Here, we provide examples of how the regulation of DNA methylation, small RNAs, and NMD helps activate TIR/TNL gene expression in response to PAMPs.

Following flg22 application, the 5-methylcytosine DNA glycosylase/lyase REPRESSOR OF SILENCING 1 erases DNA methylation from the promoters of genes (such as the TNL gene *RMG1*), likely rendering the promoter region more accessible to TF binding (Yu et al., 2013; Halter et al., 2021). DNA demethylation, particularly at the RdDM-associated CHG/CHH sequence patterns (Du et al., 2014; Johnson et al., 2014; Li et al., 2018), also occurs during SA-triggered immunity and CNL ETI (Dowen et al., 2012; Yu et al., 2013).

The grip of small RNAs on Arabidopsis TNL transcript accumulation (Figure 5, A and C) weakens in response to PAMPs (flg22) due to reduced *miR825* expression (López-Márquez et al., 2021). This is in line with the established roles of conserved miRNAs miR482, miR472, and miR2118 in limiting NLR gene expression (Shivaprasad et al., 2012; Boccara et al., 2014). In another study, the autoimmunity of the TNL mutant *snc1* was associated with reduced small RNA biogenesis, leading to the widespread upregulation of NLR gene expression (Cai et al., 2018). Hence, the regulation of TNL transcript abundance likely follows a model with a feed-forward loop that is kept in check by *miRNA*s and released by PAMP (e.g. flg22) perception.

Similarly, PAMP (flg22) perception promotes the proteasomal degradation of NMD components, allowing for rapid TNL gene upregulation (Jung et al., 2020; Raxwal et al., 2020). The suppression of NMD is predicted to expose cryptic (i.e. not expressed under normal conditions) forms of truncated TIR-containing proteins (Figure 5, A and C). Such forms have been proposed to be translated from TNL transcripts with premature stop codons that would normally be eliminated via NMD (Parker et al., 2021).

Taken together, these recent findings highlight PAMP (flg22) perception as a kick-start to the removal of multiple brakes on TNL/TIR gene expression to enable the rapid engagement of this immunity barrier (Figures 2A and 5C).

## Concluding remarks

We have attempted to provide an integrated view of molecular events in plant TIR immunity signaling, from its initiation to defense execution and pathogen resistance. Emerging themes are the biochemical versatility of TIR domains and the potential for self-amplification of TIR signaling once its components are released by PAMP and/or effector perception. Another emerging insight is the myriad of transcriptional and posttranscriptional mechanisms used to constrain this essential but ultimately dangerous process to avoid physiological fitness costs. An unresolved question is at what level TIR/TNL signaling and TIR-generated molecules play roles in balancing plant responses to biotic and abiotic stresses encountered in the terrestrial environment. Increased knowledge of how plants fine-tune their stress pathways in nature is of fundamental interest and important for the biotechnological improvement of crop performance.

One of the major challenges in developing a coherent model of plant TIR signaling is to align the newly characterized enzymology with the domain arrangements of full-length proteins and TIR subtypes conferring potentially different stress-triggered outcomes. While EDS1–RNL complexes are central mediators of TNL receptor and TIR protein-triggered host defense and cell death in seed plants, it remains unclear whether these modules are activated directly by specific TIR-generated small molecules. The observed *EDS1-*independence of certain TIR-induced cell death responses in plants suggests that TIRs might either simply deplete NAD^+^ or collectively produce a cocktail of small molecules, perhaps only some of which are perceived as signals by EDS1 family–RNL modules for mobilizing Ca^2+^-based immunity cascades. This notion is supported by the fact that nonseed plants have multiple TIR-containing proteins but no EDS1 family members. Further study of the occurrence and activities of immunity modules during plant evolution should provide a clearer picture of how plant defense network architectures are built and elaborated on in response to pathogen attack.

## Accession numbers

Source data and code to reproduce the plots, as well as the results of homology-based modeling of TNPs, are available at https://github.com/rittersporn/Lapin-etal_PlantCell-review_2022.

## Notes

D.L., O.J., J.E.P.: conceptualization; D.L., O.J.: analysis of raw and processed RNA-seq and ChIP-seq data, domain annotation; D.L.: preparation of GitHub repository; D.L., O.J., Z.W.: preparation of figures; all authors: writing, editing, reviewing; D.L., X.L., J.E.P.: supervision.

The authors responsible for distribution of materials integral to the findings presented in this article in accordance with the policy described in the Instructions for Authors (https://academic.oup.com/plcell) are: Dmitry Lapin (d.lapin@uu.nl) and Jane E. Parker (parker@mpipz.mpg.de).
